# Impact of High-Risk Mutations and Treatment Intensity in Accelerated-Phase Blast-Phase MPN Without Adverse-Risk Karyotype and TP53

**DOI:** 10.3390/curroncol33070419

**Published:** 2026-07-12

**Authors:** Verna Cheung, Marta Davidson, Eshetu G. Atenafu, Andrea Arruda, Jaime O. Claudio, Aniket Bankar, Dawn Maze, Vikas Gupta, Hassan Sibai

**Affiliations:** 1 Princess Margaret Cancer Centre, Toronto, ON M5G 2M9, Canadajaime.claudio@uhn.ca (J.O.C.);; 2 Lawrence S. Bloomberg Faculty of Nursing, University of Toronto, Toronto, ON M5T 1P8, Canada; 3 Department of Biostatistics, University Health Network, Toronto, ON M5G 1X6, Canada; eshetu.atenafu@uhn.ca; 4 Division of Biostatistics, DLSPH, University of Toronto, Toronto, ON M5T 3M7, Canada

**Keywords:** high-risk mutations, treatment intensity, myeloproliferative neoplasm

## Abstract

The prognostic significance of myelodysplasia-related gene mutations (MDS-RGMs), defined by the ELN 2022 classification and Mutation-Enhanced International Prognostic Score System high-risk mutations (MIPSS70-HRM), in accelerated-phase (AP) and blast-phase (BP) myeloproliferative neoplasms (MPNs) remains unclear. We conducted a retrospective study of 101 AP/BP MPN patients with intermediate-risk cytogenetics, excluding TP53 mutations and adverse-risk karyotypes. We evaluated whether MDS-RGM or MIPSS70-HRM predicted treatment response, overall survival (OS), or disease-free survival (DFS) and whether treatment intensity affected OS. Patients included AP (29.7%) and BP (70.3%), with 55% receiving intensive therapy. Allogeneic stem cell transplant (ASCT) significantly improved OS (HR 0.31, 95% CI 0.19–0.50; *p* < 0.0001), as did AP versus BP at transformation (HR: 0.43, 95% CI 0.26–0.73; *p* = 0.0016). Among patients ≤ 70 years with ECOG 0–1 (*N* = 77), ASCT and reversion to chronic-phase MPN (cMPN) were associated with longer OS (*p* < 0.0001 and *p* = 0.0475). Treatment intensity alone did not significantly affect OS (*p* = 0.1991).

## Simple Summary

This retrospective study examined 101 patients with advanced myeloproliferative neoplasms (MPNs) in the accelerated phase or blast phase to determine whether certain high-risk genetic mutations, defined by the ELN 2022 classification (MDS-RGM) and the MIPSS70 prognostic system (MIPSS70-HRM), could predict treatment response or survival outcomes. This study revealed neither MDS-RGM nor MIPSS70-HRM clearly predicted treatment response, overall survival, or disease-free survival. Instead, the most important factor associated with improved survival was undergoing an allogeneic stem cell transplant. Patients with accelerated phase rather than blast phase also had better survival outcomes. Among younger and fitter patients (70 years or younger with good performance status), both ASCT and achieving a return to the chronic phase of MPN were associated with longer survival. In contrast, the intensity of treatment alone did not significantly improve overall survival. Overall, the study suggests that stem cell transplantation and disease stage at transformation are more important predictors of survival than the specific high-risk mutation groups examined.

## 1. Introduction

Myeloproliferative neoplasms (MPNs) are a group of rare clonal disorders of hematopoietic progenitor cells associated with disease-related symptoms, thrombotic events, and risk of transformation to acute myeloid leukemia [1,2,3,4]. In MPN, as disease progresses to accelerated phase (AP) and blast phase (BP), especially BP MPN, prognosis and survival outcomes become poor [1,2,3,4]. The presence of an adverse-risk karyotype and *TP53* mutations (*TP53^Mut^*) often hold dismal outcomes in MPN [1,2,3,4]. Davidson et al. (2024), through a single-center retrospective analysis, examined response outcomes of treatment intensity in 138 patients with AP/B MPN [2]. Davidson et al.’s (2024) retrospective analysis has demonstrated that, although intensive treatments yield higher responses rates than non-intensive treatment, more patients in the study reverted to chronic-phase MPN (cMPN) instead of completed remission (CR), 66% vs. 30% respectively [2]. Furthermore, patients with *TP53^Mut^* and RAS pathway mutation also demonstrated inferior response to intensive treatment [2]. In addition, the benefits of intensive therapy are often limited to patients who have good performance status and are able to undergo an allogenic hematopoietic stem cell transplant (ASCT) [1,2]. Given these limitations in treatment response and the poor prognostic impact of specific genetic alterations, there is a need to refine molecular risk stratification frameworks in AP/BP MPN. Through better understanding of how certain molecular risks may impact response to intensity of blast reduction therapies and in turn survival outcome, more tailored treatments for patients with AP/BP MPN can be developed.

In 2022, the European Leukemia Net (ELN) guidelines for diagnosis and treatment of AML were updated to also include myelodysplasia-related gene mutation (MDS-RGM) and it impact on diagnosis and treatment [5]. However, the impact of the ELN 2022 definition of MDS-RGM on MPN AP/BP is not well understood. By re-examining the existing data from Davidson et al. [2] we aimed to determine if the ELN 2022 MDS-RGM or Mutation-Enhanced International Prognostic Score System high-risk mutations (MIPSS70-HRM) is a better predictor of response to intensive and non-intensive treatment, disease-free survival (DFS), and overall survival (OS) in *TP53* wildtype (*TP53^WT^*) with non-adverse-risk karyotype AP/BP MPN. From this point forward, MDS-RGM may be used interchangeably with ELN 2022 MDS-RGM.

## 2. Material and Method

We performed a retrospective analysis on patients with MPN-AP/BP treated at our center in the last two decades [2]. Analysis excluded patients with ELN-2022-defined adverse-risk karyotype and *TP53^Mut^*, given both are known for poor prognosis and response to blast-reductive treatments [1,2]. Response criteria defined as per Princess Margaret criteria were complete remission (CR); complete remission with incomplete count recovery (CRi)—no circulating blast or BM blast < 5%, platelets < 100 × 10^9^/L or ANC < 1 × 10^9^/L; reversion to chronic MPN (cMPN)—peripheral blood (PB) blast < 10% or BM blast 5–9% or BM blasts < 5% and MPN feature on BM biopsy; progressive disease (PD)—increase in PB/BM blast ≥ 50% or new extramedullary disease; and early death—which is defined as death within 60 days of treatment initiation [2].

The mutations considered high-risk in the ELN 2022 MDS-RGM include *ASXL1*, *BCOR*, *EZH2*, *RUNX1*, *SF3B1*, *SRSF2*, *STAG2*, *U2AF1*, and *ZRSR2*. In MIPSS70-HRM the high-risk mutations are: *ASXL1*, *EZH2*, *SRSF2*, *IDH1*, *IDH2*, and *U2AF1*. Testing for these mutations was completed at time of disease progression, using the University Health Network Hematological Malignancies Panel (UHN-HMP) v3.0. These mutations had to be classified as pathogenic or likely pathogenic. Patients with such mutations but classified as variants of unknown significance were not considered to have an HRM. Unfortunately, serial molecular testing was not available for all patients.

This retrospective analysis was approved by the UHN Research Ethics Board (REB CAPCR 16-5169).

### Statistical Analysis

Categorical variables such as gender, type of treatment, ECOG, MPN transformation type and treatment intensity were presented as counts and percentages. Continuous variables such as age, WBC, as well as follow-up were presented as medians with ranges/interquartile range (IQR). Chi square and/or Fisher’s exact test (as appropriate) was used to assess the association of categorical covariates of interest on best response while a Kruskal–Wallis test was used to assess the relationship of continuous covariates of interest with best response. The main outcome variable of interest includes time to death. Time to death was calculated in months from the date of disease transformation to the date of death or to the last follow-up, whichever comes first. Time to relapse was calculated in months from the date of disease transformation to the date of relapse or date of death or to the last follow-up, whichever comes first. The OS and DFS rates were calculated using the Kaplan–Meier product-limit method and a log-rank test was used to assess the impact of covariates of interest on survival curves. All *p*-values were 2-sided and, for the statistical analyses, *p* < 0.05 was considered to indicate a significantly different result. Statistical analyses were performed using SAS version 9.4 of the SAS system for Windows (Copyright © 2023 by SAS Institute, Inc., Cary, NC. USA).

## 3. Results

### 3.1. Study Population Characteristics and Outcomes

Of 148 MPN-AP/BP patients, *N* = 101 patients met the inclusion criteria. The median age at diagnosis was 65 years (range: 36 to 87 years of age), with an IQR of 56 to 70 years of age. The cohort included *N* = 41 females (40.6%) and N = 60 males (59.4%). All patients had a previous cMPN diagnosis (polycythemia vera or essential thrombocytosis 18.8%, primary or secondary myelofibrosis 63.4%, other subtypes of MPN 17.8%) (Table 1).

Regarding disease phase at transformation, 30 patients (29.7%) had AP MPN and 71 (70.3%) had BP MPN. In terms of intensity of treatment, N = 46 (45.5%) patients received non-intensive treatment, and *N* = 55 (54.5%) patients received intensive treatment. Forty-one patients (40.6%) underwent ASCT, while N = 60 patients (59.4%) did not (herein referred to as non-ASCT).

Patients received one of four treatment regimens: induction chemotherapy 3 + 7 (*n* = 24, 23.8%), FLAG/NOVE (*n* = 31, 30.7%), hypomethylating agents alone (HMA; *n* = 33, 32.7%), or HMA combined with venetoclax (HMA + VEN; *n* = 13, 12.9%) (Table 1). The HMA-based regimens were non-intensive therapies, and 3 + 7 and FLAG/NOVE were considered intensive therapies. CR/CRi, cMPN, PD and early death as best response to therapy were seen in 38.6%, 37.6%, 17.8%, and 5.9%, respectively (Table 1). We consider CR/CRi and cMPN as responders to treatment and PD and early death as non-responders.

### 3.2. Univariate Analysis for OS

**Figure 1:**
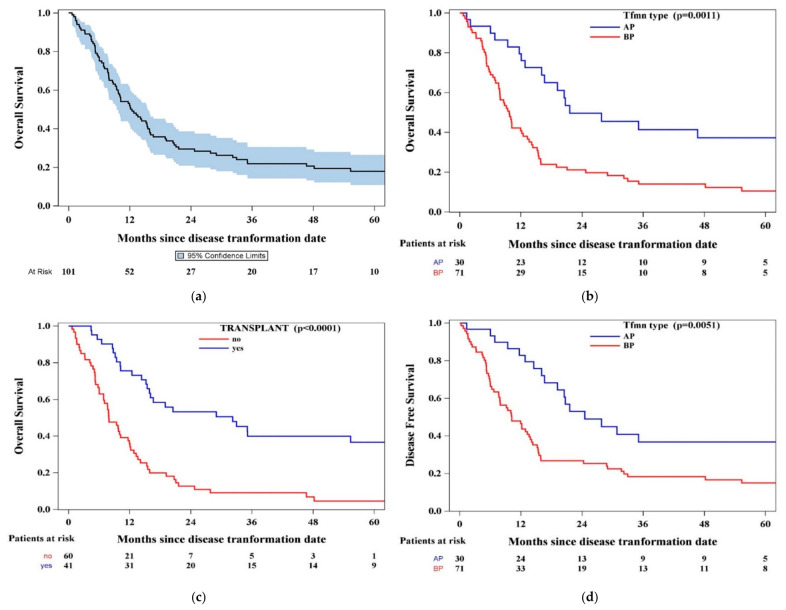
(**a**) OS distribution from date of disease transformation; (**b**) OS distribution by type of MPN transformation; (**c**) OS distribution by transplant; (**d**) DFS distribution by type of MPN transformation.

Median OS for the entire cohort was 12.5 months (95% confidence interval (CI): 9.4–15.6). The 12-month and 24-month OSs were 52% (95% CI: 42–61%) and 30% (95% CI: 21–39%), respectively (Figure 1a).

Univariate analysis for OS revealed that the type of MPN transformation (AP vs. BP), allogeneic hematopoietic stem cell transplant (ASCT), and ECOG were predictive of OS. Patients with AP showed longer median OS compared to patients in BP (21.6 months vs. 9.73 months) (Figure 1b) as did patients who had undergone ASCT compared to those who did not (32.2 months vs. 7.9 months) (Figure 1c). DFS was also longer in AP patients compared to BP patients (24.5 months vs. 10.2 months) (Figure 1d). In this entire cohort of patients (*N* = 101), analyzing the OS at 90-day, 180-day, and 270-day intervals between ASCT vs. non-ASCT patients, the *p*-value remains statistically significant and patients who proceeded with ASCT had better OS (Supplementary Table S1).

In the entire cohort, better ECOG (0 vs. 1) was associated with better OS (*p* = 0.004) (Table 2). Interestingly, there was no significant difference in OS between patients with or without ELN 2022 MDS-RGM (present vs. absent hazard ratio (HR) = 1.28 (95% CI: 0.79–2.1; *p* = 0.318)) (Table 2). The number of mutations in general was not associated with OS with mutation > 4 HR = 1.23 (95% CI: 0.75–2.01; *p* = 0.415) (Table 2). The number of MDS-RGMs also had no significant impact on OS with mutation 1 or more HR = 1.65 (95% CI: 0.96–2.83; *p* = 0.0717) (Table 2).

Covariate analysis between treatment intensity and best response revealed that patients who received intensive therapy achieved a higher proportion of CR/CRi compared to those treated with non-intensive therapy (56.4% vs. 17.4%, *p* < 0.0001). However, again, MDS-RGM absence vs. presence did not have an impact on response type (*p* = 0.374) and neither did performance status (*p* = 0.173) (Table 3).

### 3.3. Multivariable Analysis and Response Comparison

Multivariable Cox proportional hazards modeling identified MPN transformation type and ASCT status as significantly associated with DFS (AP vs. BP *HR* = 0.50 (95% CI: 0.30–0.84; *p* = 0.009)); ASCT yes vs. no, *HR* = 0.28 (95% CI: 0.17–0.46; *p* < 0.0001) (Table 4). As for the OS (AP vs. BP *HR* = 0.47 (95% CI: 0.28–0.79; *p* = 0.004)), ASCT yes vs. no *HR* = 0.32 (95% CI: 0.20–0.53; *p* < 0.0001) (Table 4), therefore favoring AP and proceeding with ASCT.

Additional covariate analysis of treatment response to type of treatment intensity was conducted within the entire cohort (*N* = 101). This revealed that with intensive treatment (*N* = 55) more patients achieved CR/CRi and cMPN (56.4% and 29.1%, respectively) vs. the non-intensive treatment group (*N* = 46; 17.4% and 47.8%, respectively; *p* < 0.0001) (Table 5). This suggests that upfront intensive treatment allows patients to achieve a deeper response compared to non-intensive treatment.

### 3.4. Analysis of Patients Eligible for ASCT

Further analysis was conducted in patients aged ≤ 70 with ECOG 0 or 1. In this otherwise fit for ASCT subgroup, *N* = 24 received non-intensive treatment and *N* = 54 received intensive treatment. Again, in the intensive group, more patients achieved CR/CRi and cMPN (55.6% and 29.6%, respectively) vs. non-intensive (20.8% and 54.2% respectively, *p* = 0.0215) which is statistically significant (Table 6). The intensity of treatment (intensive vs. non-intensive) did have some difference in median OS (11.1 months vs. 16.1 months, *p* = 0.1991), but it was not statistically significant. Further analysis in this subgroup based on patients’ best response to treatment and having either ECOG 0 or 1 did not show a significant impact on response in this subgroup (*p* = 0.0773) (Table 6).

**Figure 2:**
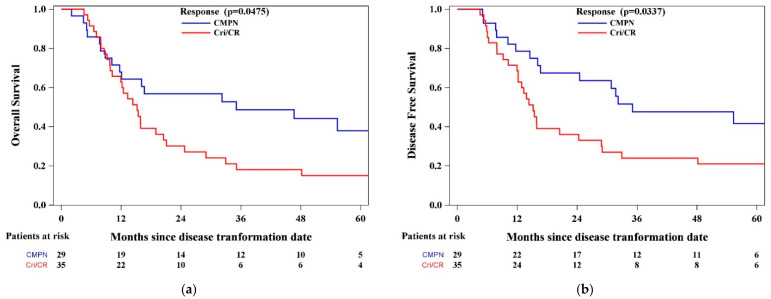

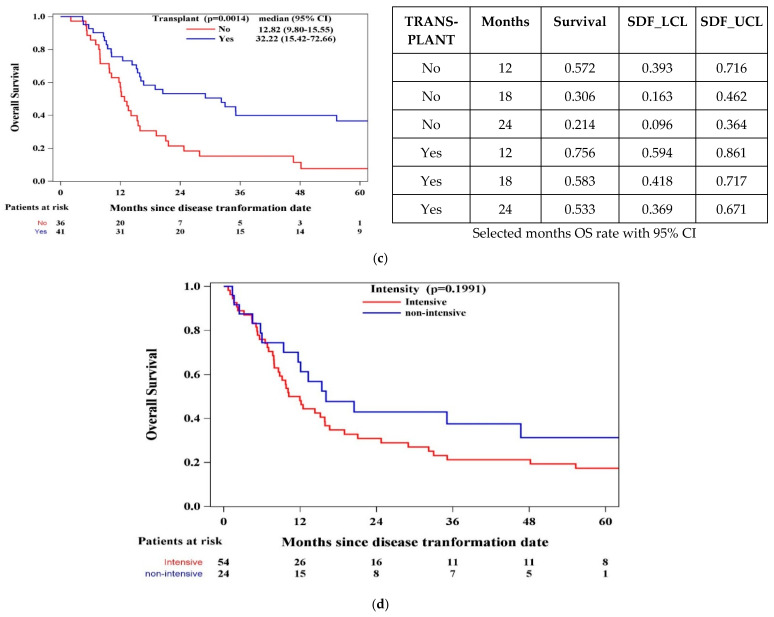
(**a**) OS distribution by response type, limited to ECOG 0–1, age ≤ 70; (**b**) DFS distribution by response type, limited to ECOG 0–1, age ≤ 70; (**c**) OS distribution OS in months for ECOG ≤ 1 and age ≤ 70 and transplant (ASCT), with table showing the selected months’ OS rate with 95% CI; (**d**) OS distribution limited to ECOG 0 and 1 age ≤ 70, by treatment intensity.

However, reversion to cMPN (*N* = 29) showed better median OS compared to those in CR/CRi (*N* = 35) (35.1 months vs. 15.2 months, *p* = 0.0475) (Figure 2a). Median DFS was also better in cMPN compared to those in CR/CRi (35.1 months vs. 15.2 months, *p* = 0.0337) (Figure 2b). Within this subgroup, *N* = 41 underwent ASCT and *N* = 36 non-ASCT, and median OS was both numerically and statistically significantly different (32.2 months vs. 12.8 months, *p* < 0.0014) (Figure 2c). However, in further analysis based on treatment intensity (intensive vs. non-intensive) as blast reduction therapy prior to proceeding with ASCT, this did not have a significant impact on OS (*p* = 0.1991) (Figure 2d). Examining the OS at 90-day intervals between ASCT vs. non-ASCT patients, the *p*-value was not significant between the two groups of patients at the 90-day, 180-day, and 270-day intervals (Supplementary Table S2). However, as Figure 2c indicates, beyond the 12-month timeframe, the OS showed both numerically and statistically significant differences between the two groups and OS was better for those who underwent ASCT (*p* = 0.0014).

Focusing on only patients that have responded (cMPN and CR/CRi, *N* = 77) and comparing their treatment regimens (intensive vs. non-intensive), intensive treatment produced more responders (*N* = 47) compared to non-intensive treatment (*N* = 30). Secondly, among responders that underwent intensive therapy numerically more underwent ASCT (*N* = 28) compared to non-responders that underwent non-intensive treatment (*N* = 13). Interestingly, among the group of responders who received intensive treatment, average survival time was 35.8 months compared to responders who received non-intensive treatment; the average survival time was 24.4 months. However, when ASCT patients were excluded, the average survival time among the intensive treatment group (*N* = 19) was 16.2 months, with *N* = 2 having AP and *N* = 17 having BP MPN. For responders in the non-intensive treatment group (*N* = 17), which comprised mostly cMPN patients, the average survival time was 20.1 months. Within this non-intensive group, the non-ASCT group, *N* = 10 had AP and *N* = 7 had BP MPN.

### 3.5. Comparing ELN 2022 to MIPSS70 Mutation

The ELN 2022 [5] and MIPSS70 [6] recognize different mutations as high-risk (Table 7). In the entire cohort of *N* = 101 patients, 21 patients were missing, as molecular testing was not available at the time of disease progression. In the entire cohort the frequencies of HRMs were as follows: *ASXL1* (20.8%), *BCOR* (4.9%), *EZH2* (8.9%), *RUNX1* (12.9%), *SF3B1* (2%), *SRSF2* (19.8%), *STAG2* (10.9%), *U2AF1* (4.9%), *IDH1* (7.9%), *IDH2* (11.9%), *ZRSR2* (4.9%). We conducted further analysis based on the presence of HRMs to see if this may have an impact on treatment response, OS, and/or DFS in the entire cohort (*N* = 101) (Supplementary Figures S1a–d and S2a–d). Analyzing the number of HRMs based on MIPSS70-HRM vs. ELN 2022 MDS-RGM revealed that there was no statistical significance to OS (*p* = 0.372 and *p* = 0.106) (Supplementary Figures S1a and S2b) and/or DFS (*p* = 0.359 and *p* = 0.160) (Supplementary Figure S1c,d). Multivariable analysis on the effect of treatment intensity and number of HRMs on OS (Supplementary Figure S2a,b) and DFS (Supplementary Figure S2c,d) for both MIPSS70-HRM and MDS-RGM was also not statistically significant. Supplementary Table S3 also compares the number of HRMs based on MDS-RGM vs. MIPSS70-HRM distributed by intensity of treatment and shows numerically no significant difference in distribution. Further analyses of the specific mutations and survival outcomes and/or treatment response were also carried out and again none of the specific mutations showed any statistically significant correlation to survival outcomes or treatment response (graphs not provided).

## 4. Discussion

MPN in AP/BP carries a dismal prognosis especially with the presence of an adverse-risk karyotype and/or *TP53^Mut^* [1,2,3,4,7,8,9,10]. To date, ASCT remains the only procedure associated with durable and improved OS compared to other systemic therapies alone [7,8,9,10]. The unique perspective this retrospective analysis provides is that we are examining an MPN population that does not have such poor prognostic factors—patients with *TP53^WT^* and non-adverse-risk karyotypes—and whether ELN 2022 MDS-RGM or MIPSS70-HRM is a better predictor of treatment response and overall survival outcomes in this context. Secondly, it also examines in this patient population what the best approach might be in terms of choice of blast reductive therapy to improve outcomes.

In our retrospective analysis, the median OS for the entire cohort was 12.5 months, which is similar to existing literature [2,7,8]. In addition, both the entire cohort and the subgroup of patients of those who underwent ASCT had better OS (Figure 1c and Figure 2c), and patients in AP MPN had a more favorable OS and DFS (Figure 1b,d, and Table 2). These findings again aligned with the current literature and clinical practice, whereby the preference is to initiate treatment in intermediate- or high-risk-disease patients with cMPN or AP MPN and/or to push forward with aggressive treatment such as ASCT, especially before progression to BP MPN, given the survival outcomes and prognosis are less optimal when disease progresses to BP MPN [1,2,3,4,7,8,9,10]. However, we do acknowledge the potential for selection bias, whereby patients undergoing ASCT are generally younger, have better performance status, and are more likely to achieve disease control before transplantation, which may have contributed to improved outcomes independent of ASCT itself.

However, what remains debatable is the intensity of blast reduction therapies to be used as the agent to bridge a patient in AP/BP MPN to ASCT and also the necessity of achieving CR/CRi vs. cMPN prior to proceeding with ASCT [7,8,9,10]. In BP MPN, intensive treatments such as 3 + 7 or FLAG-IDA were used to achieve CR/Cri [7,8]. Several studies also report that patients who achieve CR/CRi prior to proceeding with ASCT have improved outcomes [7,9,11,12]. However, a retrospective study by Gagelmann et al. demonstrated that the 5-year survival rate was 65% for patients with AP MPN who proceed with ASCT without bridging therapy, compared to 64% for patient with cMPN; however, AP MPN had a higher relapse rate compared to cMPN patients [13]. There is also to date no clear consensus on the necessity of bridging therapy in AP MPN prior to ASCT [8,13]. Other studies have also demonstrated that it is not disease status at ASCT but the presence or absence of complex/adverse-risk karyotypes and high-risk mutations that were predictive of inferior outcomes post ASCT [8,10,14]. Tefferi et al. (2023) suggest that, for patients with complex/adverse-risk karyotype and high-risk mutations in AP MPN, depending on the blast percentage, bridging therapy prior to ASCT may be appropriate as the outcomes are often dismal if the patient progresses to BP MPN [8]. But Tefferi et al. also mention the inclination to proceed directly to ASCT in AP MPN if blast burden is low [8]. However, it is worth noting that in our retrospective analysis we did not find any significant associations between the presence of adverse mutations and impact on response to treatment, OS and/or DFS, but we acknowledge that this may be due to the smaller sample size of the study.

**Figure 3:**
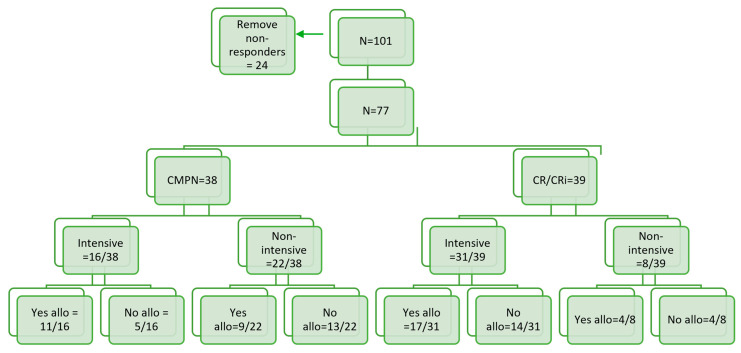
Responder outcomes flow chart.

Interestingly, in this retrospective analysis, in the subgroup of patients with ECOG 0 or 1 and age eligible for ASCT (age ≤ 70) with AP/BP MPN, those with reversion to cMPN had a significantly better OS and DFS compared to those who achieved CR/CRi (Figure 2a,b). The survival difference between cMPN and CR/CRi groups became evident after 12 months. It is also noteworthy that, although numerically more patients achieved cMPN or CR/CRi with intensive treatment, OSs between intensive vs. non-intensive treatment in this otherwise fit group of patients were not statistically significantly different (Figure 2d, *p* = 0.1991), thus adding to the debate about the need to achieve deeper remission for better outcomes. A possible explanation for this difference may be the treatment toxicities and complications that can occur with more intensive treatment regimens which are recognized in both clinical practice and the literature [1,2,3,4,5,7,15]. In our study, although intensive treatment produced more responders (those who achieve CR/CRi or cMPN) compared to patients in the non-intensive treatment group, when we excluded patients who proceeded to ASCT, it was the non-intensive treatment group that had a better average survival time. In our retrospective analysis of patients achieving CR/CRi with intensive therapy, 14/31 (45.2%), but did not proceed to ASCT and received only a limited amount of consolidation therapy. However, among patients reverting to cMPN following non-intensive therapy, 13/22 (59.1%) continued with non-intensive treatment as long as they were deriving benefit. Similarly, Patel et al. (2024) also noted that, although patients who underwent induction type blast reductive therapies achieved higher CR/CRi compared to those who received less intensive (i.e., HMA-based) regimens, there was no significant impact on OS between the two groups of patients [16]. It is also noteworthy, in Patel et al., that patients who received HMA-based therapy also had numerically longer OS [16]. Patel et al.’s findings are similar to the findings of this retrospective analysis and also suggest that perhaps it is the ability to continue non-intensive therapy such as HMA-based treatment and ongoing exposure to cytoreductive therapy that may have contributed to better OS and DFS in the cMPN group, despite not achieving deeper remission upfront [2,16]. The authors, however, do acknowledge that in the non-intensive with reversion to cMPN patient group, more of these patients were in AP MPN, which may have had a positive impact leading to a better average survival time (Figure 3 and Table 8).

Another reason the use of intensive treatment remains debatable is due to the presence and availability of novel non-intensive treatment modalities and also a lack of prospective controlled studies in this area [7,8,10,13,14]. Non-intensive treatment, at present, often involves HMA-based combination regimens such as HMA + VEN [7,8,16], and studies show HMA-based therapies have yielded 20–43% CR/CRi, with median OS of 4 months–1 year [2,7,8,16]. Furthermore, even in patients with poor prognostic factors such as adverse-risk karyotype and/or *TP53^Mut^*, combination therapies with HMA + VEN as a backbone have shown improved response rate and allowed for the patients, otherwise unfit for intensive blast reductive treatments such as induction chemotherapy, to safely receive blast reductive therapy as a bridge to ASCT [3,4,14,15,16,17]. In addition, non-intensive treatments are often able to be given on an outpatient basis, which is not only more cost-effective but also improves timeliness to initiating treatment as it does not require hospital admission, which is dependent on hospital bed and staffing availability.

Secondly, the emergence of maintenance therapies such as oral azacitidine (OA) has improved OS and relapse-free survival for acute myeloid leukemia patients otherwise unfit to proceed with ASCT [18]. Although, in our retrospective study, none of our patients received OA, this is a strategy that also needs to be explored to optimize survival outcomes in AP/BP MPN otherwise unfit and/or ineligible for ASCT.

In addition, with better access to advanced genomic and molecular testing, there is also an increased interest in the role of molecular and genetic profiles to assess treatment response with non-intensive therapy [7,8,9,19,20]. These studies have noted that the presence of adverse-risk karyotypes and RAS pathway mutations may be predictors of inferior response to HMA + VEN in both BP-MPN and AML, and the presence of *IDH1/2* may predict a more favorable response, meaning achievement of CR/CRi [19,20]. For instance, Shaw et al.’s (2025) retrospective study noted a significantly higher rates of CR/CRi in patients with *IDH1* mutations (OR = 0.16, 95% CI: 0.03–0.9, *p* = 0.04) despite none of the patients in the study receiving an *IDH1* inhibitor as frontline therapy [20]. However, Shaw et al. also noted that there was no specific adverse molecular mutation that was associated with failure to achieve CR/CRi [20]. In our retrospective analysis, we also examined the impact of HRMs in AP/BP MPN with treatment intensity and response. However, multivariate analysis did not show any significant findings (Supplementary Figure S2a–d). Nor did the number of HRMs based on MIPSS70-HRM vs. ELN 2022 MDS-RGM have an impact on OS and DFS (Supplementary Figure S1a–d). Further analysis examining the specific HRMs also did not reveal any correlation or significant impact on OS, DFS, and/or impact on treatment response. The authors do acknowledge that the small sample size of this study, and hence a smaller portion of patients with HRMs, may account for the lack of power to detect statistical significance and/or impact of HRMs on OS, DFS and treatment response in our particular study. But, Shaw et al. also acknowledge that AP/BP MPN is a different entity compared to de novo AML, and more comprehensive risk stratification tools tailored for AP/BP MPN are needed. However, to date, the consensus in the literature remains that ASCT is the only treatment that provides the most meaningful and prolonged OS and DFS in patients who have progressed to AP/BP MPN [7,8,9,20].

By excluding patients with an adverse-risk karyotype and *TP53*^Mut^, this retrospective analysis offers a unique perspective on selection of treatment intensity and its impact on survival outcomes in AP/BP MPN without ultra-high-risk molecular features. Consistent with the existing literature, ASCT remains the only therapeutic approach associated with a substantial and durable improvement in OS and DFS [1,2,7,8,9,10,11]. Furthermore, within the AP/BP MPN population, established prognostic models—both ELN-2022 MDS-RGM and MIPSS70-HRM—did not demonstrate predictive value for OS, DFS, or treatment response in this cohort. This finding suggests that, in AP/BP MPN, the prognostic relevance of these molecular classifiers may be attenuated, potentially due to the overriding impact of disease biology at transformation.

Interestingly, our data highlight a nuanced outcome among patients who did not proceed to ASCT despite being otherwise fit and eligible. In this subgroup, patients who achieved reversion to cMPN experienced improved OS compared to those who achieved CR/CRi (OS 35.1 months vs. 15.2 months, respectively, *p* = 0.0475). This observation raises important questions regarding the biological and clinical significance of disease state definitions in this setting and whether reversion to cMPN may represent a more stable or therapeutically meaningful endpoint than conventional remission criteria in the absence of transplant.

Secondly, although intensive treatment (i.e., induction chemotherapy) resulted in higher initial response rates, this did not translate into a survival advantage among patients who did not undergo ASCT. In other words, treatment intensity alone was insufficient to improve long-term outcomes, underscoring the limited long-term durable responses with intensive chemotherapy alone.

### Limitations

The authors do acknowledge there are limitations to this study. Firstly, this was a retrospective analysis, with patient data spanning across close to two decades; during this time there have been changes to treatment regimens and emergence of novel agents that have changed survival outcomes. One such treatment is venetoclax, which started to be incorporated into HMA treatment around 2020. This treatment heterogeneity related to the long study period is a limitation of this retrospective analysis. Secondly, the availability and use of supportive treatments such as antifungals and antibiotics during patient nadir also has had an impact on survival outcomes, which has had an impact on results of this study. In the past two decades, there have been advances in genomic and molecular testing. Unfortunately, such tests were not available at the time of initial MPN diagnosis for all patients, and most genomic and molecular testing was completed at the time of disease progression only. Having sequential genomic and molecular data might have provided additional information regarding clonal evolution which can impact the disease trajectory.

## 5. Conclusions

As our study has demonstrated, neither MDS-RGM nor MIPSS70-HRM was predictive of treatment response or survival in AP/BP MPN. Instead, outcomes were primarily determined by disease phase at transformation and access to ASCT.

Interestingly, our retrospective analysis suggests that, in the absence of ASCT, even for patients ≤ 70 years of age with good performance status, intensity of treatment did not translate into better survival outcomes. Notably, non-intensive treatment did not seem to show inferiority to intensive treatment regimens in AP/BP MPN. However, to date what remains unclear is the optimal number of non-intensive cycles before treatment escalation, particularly for transplant-eligible patients who do not achieve response after two or three cycles of non-intensive therapies.

Taken together, these observations might suggest that the use of standard intensive induction type regimens in AP/BP MPN should be carefully individualized, and in the era of emerging novel agents that offer less intensive blast reduction treatment regimens, they further underscore the urgent need for prospective studies to define the role of various treatment regimens as a bridge to ASCT in this setting.

## Figures and Tables

**Table 1:** Patient characteristics and outcomes.

	Total (N = 101)
Age	
N	101
Median (range)	65.0 (36.0, 87.0)
IQR	56.0, 70.0
Gender, *n* (%)	
Female	41 (40.6%)
Male	60 (59.4%)
Transplant, *n* (%)	
No	60 (59.4%)
Yes	41 (40.6%)
MPN transformation type (Tfmntype), *n* (%)	
AP	30 (29.7%)
BP	71 (70.3%)
Previous types of cMPN	
N	101
Polycythemia vera/Essential thrombocythemia	19 (18.8%)
Primary/Secondary myelofibrosis	64 (63.4%)
Other subtypes of MPN	18 (17.8%)
Treatment group, *n* (%)	
3 + 7	24 (23.8%)
FLAG/NOVE	31 (30.7%)
HMA	33 (32.7%)
HMA + VEN	13 (12.9%)
Best response, *n* (%)	
Early death	6 (5.9%)
CMPN	38 (37.6%)
PD	18 (17.8%)
Cri/CR	39 (38.6%)
Status at last follow-up, *n* (%)	
Alive	19 (18.8%)
Dead	82 (81.2%)

**Table 2:** Univariate analysis for OS using COX proportional hazards model.

Parameter	Level	Overall *p*-Value	*p*-Value	HR (95% CI)
ECOG (Ref = 0)	1	0.0101	0.0037	2.32 (1.31–4.09)
	2		0.5144	0.63 (0.15–2.56)
Age		0.0287	0.0287	1.02 (1.00–1.05)
Sex	F	0.5912	0.5912	0.89 (0.57–1.38)
Number of high-risk mutations	1 or more	0.0717	0.0717	1.65 (0.96–2.83)
Transplant	Yes	<0.0001	<0.0001	0.31 (0.19–0.50)
Type of MPN transformation AP/BP	AP	0.0016	0.0016	0.43 (0.26–0.73)
MDS-RGM	Present	0.3175	0.3175	1.28 (0.79–2.09)
Number of positive mutations NGS	>=4	0.4152	0.4152	1.23 (0.75–2.01)
Treatment intensity	Non-intensive	0.9231	0.9231	1.02 (0.66–1.59)
WBC		0.1313	0.1313	1.00 (1.00–1.01)
MPN2 at diagnosis (Ref = MF)	Other	0.6343	0.5187	0.82 (0.46–1.49)
	PV/ET		0.4086	0.78 (0.44–1.39)
Fibrosis	no	0.6566	0.6566	0.90 (0.57–1.43)

**Table 3:** Distribution of covariates of interest by Best Response.

	Best Response				
	Early Death (N = 6)	CMPN (N = 38)	PD (N = 18)	Cri/CR (N = 39)	*p*-Value
Age					0.0004 ^1^
N	6	38	18	39	
Median (range)	66.5 (54.0, 69.0)	62.5 (42.0, 83.0)	72.0 (55.0, 87.0)	60.0 (36.0, 82.0)	
IQR	56.0, 68.0	54.0, 70.0	69.0, 79.0	54.0, 67.0	
WBC					0.0323 ^1^
N	6	38	18	39	
Median (range)	26.6 (1.6, 108.0)	24.2 (2.3, 145.0)	6.1 (1.0, 126.0)	9.2 (1.1, 213.0)	
IQR	7.6, 46.7	9.8, 38.8	3.4, 22.3	4.9, 23.5	
MDS-RGM, *n* (%)					0.3474 ^2^
Absent	1 (2.4%)	16 (38.1%)	6 (14.3%)	19 (45.2%)	
Present	4 (10.5%)	13 (34.2%)	8 (21.1%)	13 (34.2%)	
Missing	1	9	4	7	
Performance status, *n* (%)					0.1734 ^2^
0	4 (4.9%)	33 (40.2%)	12 (14.6%)	33 (40.2%)	
1	2 (12.5%)	4 (25.0%)	6 (37.5%)	4 (25.0%)	
2	0 (0.0%)	1 (33.3%)	0 (0.0%)	2 (66.7%)	
Treatment intensity, *n* (%)					<0.0001 ^2^
Non-intensive	2 (4.3%)	22 (47.8%)	14 (30.4%)	8 (17.4%)	
Intensive	4 (7.3%)	16 (29.1%)	4 (7.3%)	31 (56.4%)	

^1^ Kruskal–Wallis *p*-value; ^2^ Fisher Exact *p*-value.

**Table 4:** Multivariate analysis of DFS and OS.

(**a**) **Multivariable Analysis for DFS by Entire Cohort**					
Parameter	Level	*p*-Value	HR	95% CI	
MPN transformation type AP/BP	AP	0.0086	0.497	0.295	0.838
ASCT	yes	<0.0001	0.283	0.174	0.459
(**b**) **Multivariable Analysis for OS by Entire Cohort**					
Parameter	Level	*p*-Value	HR	95% CI	
MPN transformation type AP/BP	AP	0.0041	0.467	0.278	0.786
ASCT	yes	<0.0001	0.324	0.2	0.526

**Table 5:** Distribution of covariates of interest by type of treatment for entire cohort.

	Type of Treatment		
	Non-Intensive (N = 46)	Intensive (N = 55)	*p*-Value
Best Responses, *n* (%)			<0.0001 ^1^
Early death	2 (4.3%)	4 (7.3%)	
CMPN	22 (47.8%)	16 (29.1%)	
PD	14 (30.4%)	4 (7.3%)	
Cri/CR	8 (17.4%)	31 (56.4%)	

^1^ Fisher Exact *p*-value.

**Table 6:** Best response based on treatment intensity, functional status, and age.

(**a**) **Distribution of Covariates of Interest by Type of Treatment for Patients ECOG ≤ 1 and Aged ≤ 70**			
	Type of Treatment		
	Non-Intensive (N = 24)	Intensive (N = 54)	*p*-Value
Best Responses, *n* (%)			0.02151
Early death	2 (8.3%)	4 (7.4%)	
CMPN	13 (54.2%)	16 (29.6%)	
PD	4 (16.7%)	4 (7.4%)	
Cri/CR	5 (20.8%)	30 (55.6%)	
(**b**) **Distribution of Response by Type of ECOG for Patients ECOG ≤ 1 and Aged ≤ 70**			
	ECOG		
	0 (N = 70)	1 (N = 8)	*p*-Value
Best Response, *n* (%)			0.07731
Early death	4 (5.7%)	2 (25.0%)	
CMPN	27 (38.6%)	2 (25.0%)	
PD	6 (8.6%)	2 (25.0%)	
Cri/CR	33 (47.1%)	2 (25.0%)	

**Table 7:** Frequency of high-risk mutations in the entire cohort.

	Total (N = 101)
Mutations	*n* (%)
*ASXL1*	21, (20.8%)
*BCOR*	5, (4.9%)
*EZH2*	9, (8.9%)
*RUNX1*	13, (12.9%)
*SF3B1*	2, (2%)
*SRSF2*	20, (19.8%)
*STAG2*	11, (10.9%)
*U2AF1*	5, (4.9%)
*IDH1*	8, (7.9%)
*IDH2*	12, (11.9%)
*ZRSR2*	5, (4.9%)
Missing	21, (20.8%)

**Table 8:** Responder by treatment intensity and post-response treatment.

Response Treatment Intensity			ASCT	
CMPN (*n* = 38)		Total (N = 38)	Yes (N = 20)	No (N = 18)
	Non-intensive	22 (57.9%)	9 (40.9%)	13 (59.1%)
	Intensive	16 (42.1%)	11 (68.8%)	5 (31.3%)
Cri/CR (*n* = 39)		Total (N = 39)	Yes (N = 21)	No (N = 18)
	Non-intensive	8 (20.5%)	4 (50.0%)	4 (50.0%)
	Intensive	31 (79.5%)	17 (54.8%)	14 (45.2%)

## Data Availability

The original contributions presented in this study are included in the article/Supplementary Material. Further inquiries can be directed to the corresponding author.

## Supplementary Materials

The following supporting information can be downloaded at: https://www.mdpi.com/article/10.3390/curroncol33070419/s1. Figure S1: OS and DFS by high-risk mutations; Figure S2: OS and DFS by high-risk mutation and treatment intensity Table S1: Comparing transplant outcomes in first 270 days for entire cohort; Table S2: Comparing transplant outcomes in first 270 days for sub-cohort. Table S3: Comparing treatment intensity and high risk mutation distribution.
