# Supplementary material for: Impact of High-Risk Mutations and Treatment Intensity in Accelerated-Phase Blast-Phase MPN Without Adverse-Risk Karyotype and TP53
Source: Curr Oncol. 2026 Jul 12;33(7):419. doi: 10.3390/curroncol33070419 (PMC13408622; doi:10.3390/curroncol33070419)
Supplement: Supplementary file 1 [file curroncol-33-00419-s001.zip › curroncol-4330101-supplementary.pdf]

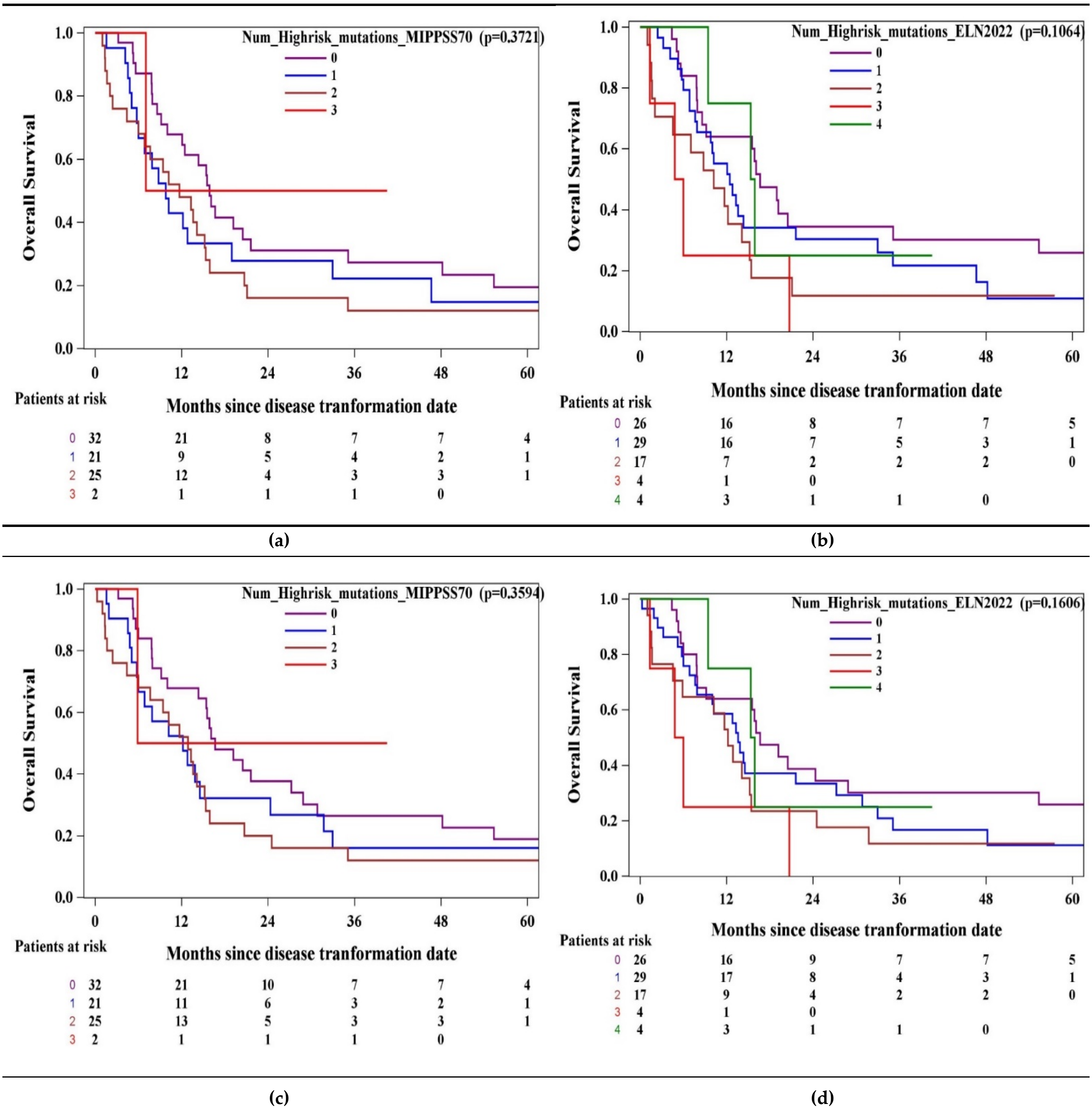

Supplementary Figure S1. OS and DFS by high-risk mutations (**a**) OS by number of high-risk mutations MIPSS70; (**b**) OS by number of high-risk mutations ELN 2022 MDS-RGM; (**c**) DFS by number of high-risk mutations MIPSS70; (**d**) DFS by number of high-risk mutations ELN 2022 MDS-RGM.

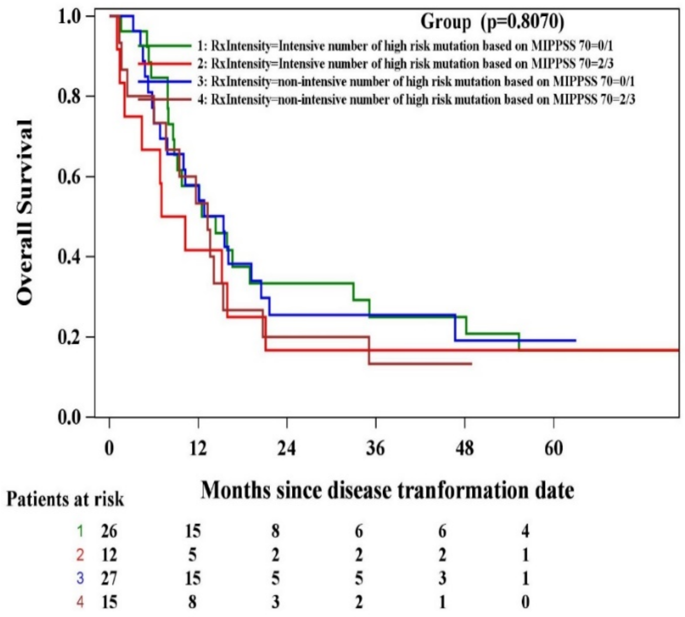

(a)

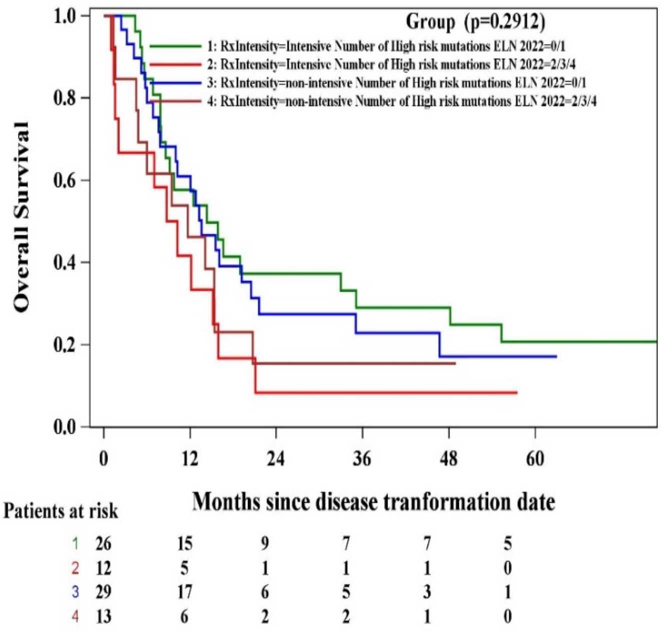

(b)

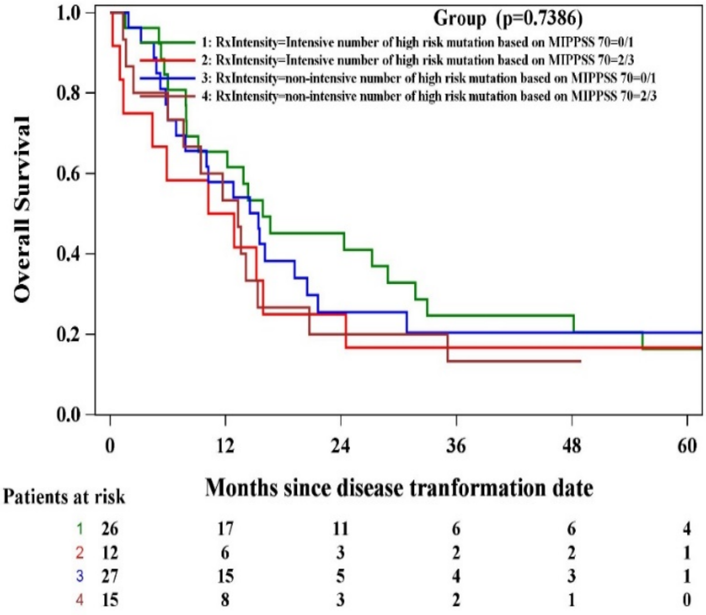

(c)

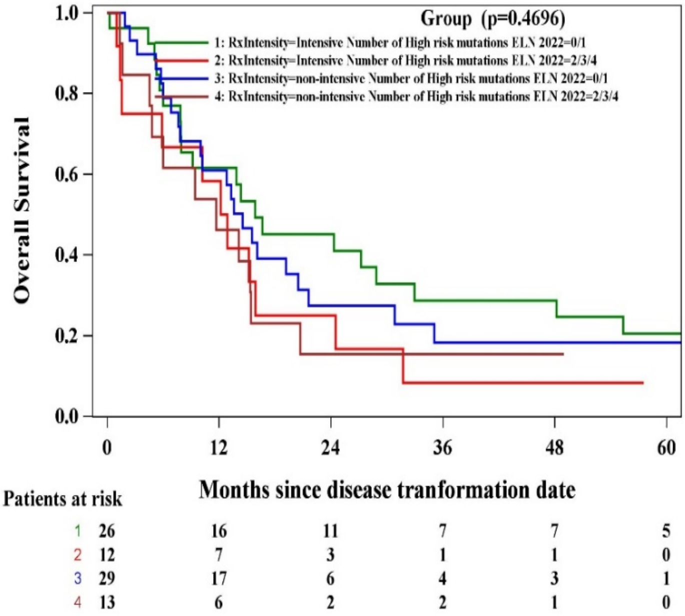

(d)

Supplementary Figure S2. OS and DFS by high-risk mutation and treatment intensity (**a**) OS by treatment type and number of high-risk mutations MIPSS70; (**b**) OS by treatment type and number of high-risk mutations ELN 2022 MDS-RGM; (**c**) DFS by

treatment type and number of high-risk mutations MIPSS70; (**d**) DFS by treatment type and number of high-risk mutations ELN 2022 MDS-RGM

Supplementary Table S1 – Comparing transplant outcomes in first 270 days for entire cohort

	TRANSPLANT		
	No (N=60)	Yes (N=41)	P-value
**Vital status at day 90**, n (%)			0.0100[1]
Alive	51 (85.0%)	41 (100.0%)	
Dead	9 (15.0%)	0 (0.0%)	
**Vital status at day 180**, n (%)			0.0017[1]
Alive	39 (66.1%)	38 (92.7%)	
Dead	20 (33.9%)	3 (7.3%)	
Missing	1	0	
**Vital status at day 270**, n (%)			0.0001[1]
Alive	28 (47.5%)	35 (85.4%)	
Dead	31 (52.5%)	6 (14.6%)	
Missing	1	0	

[1]Fisher Exact p-value;

Supplementary Table S2 Comparing transplant outcomes in first 270 days for sub-cohort

	TRANSPLANT		
	No (N=36)	Yes (N=41)	P-value
**Vital status at day 90**, n (%)			0.4675[1]
Alive	35 (97.2%)	41 (100.0%)	
Dead	1 (2.8%)	0 (0.0%)	
**Vital status at day 180**, n (%)			0.6968[1]
Alive	31 (88.6%)	38 (92.7%)	
Dead	4 (11.4%)	3 (7.3%)	
Missing	1	0	
**Vital status at day 270**, n (%)			0.1374[2]
Alive	25 (71.4%)	35 (85.4%)	
Dead	10 (28.6%)	6 (14.6%)	
Missing	1	0	

[1]Fisher Exact p-value; [2]Chi-Square p-value;

Supplementary Table S3

	type of treatment	
	non-intensive (N=46)	Intensive (N=55)
**Number of high-risk mutations ELN 2022 MDS-RGM**, n (%)		
0/1	29 (52.7%)	26 (47.3%)
2/3/4	13 (52.0%)	12 (48.0%)
Missing	4	17
**Number of high-risk mutations based on MIPPSS70-HRM** n (%)		
0/1	27 (50.9%)	26 (49.1%)
2/3	15 (55.6%)	12 (44.4%)
Missing	4	17
